# Optimum number of procedures required to achieve procedural skills competency in internal medicine residents

**DOI:** 10.1186/s12909-015-0457-4

**Published:** 2015-10-23

**Authors:** Muhammad Tariq, Nizar Bhulani, Asif Jafferani, Quratulain Naeem, Syed Ahsan, Afaq Motiwala, Jan van Dalen, Saeed Hamid

**Affiliations:** 1 Department of Medicine, Aga Khan University, Hospital, Stadium Road, P.O. Box # 3500, Karachi, Pakistan; 2 Master of Public Health Candidate, The University of Texas, Health Sciences Center at Houston, Houston, USA; 3 Department of Biological and Biomedical Sciences, Aga Khan University, Hospital, Karachi, Pakistan; 4 University of Texas, Southwestern Medical Centre at Dallas, Dallas, TX USA; 5 Communication Skills Training and Assessment, Skills Laboratory, Maastricht University, Maastricht, The Netherlands

**Keywords:** Procedural skills, Residents, Residency, Internal medicine, Competency

## Abstract

**Background:**

Procedural skills training forms an essential, yet difficult to assess, component of an Internal Medicine Residency Program. We report the development of process of documentation and assessment of procedural skills training.

**Method:**

An explanatory sequential mixed methods design was adopted where both quantitative and qualitative information was collected sequentially. A survey was conducted within the Department of Internal Medicine at The Aga Khan University Hospital, Karachi, Pakistan to determine the optimum number of procedures needed to be performed by residents at each year of residency. Respondents included both faculty and the residents in the Department. Thereafter, all responses were compiled and later scrutinized by a focus group comprising of a mix of faculty from various subspecialties and resident representatives.

**Results:**

A total of 64 responses were obtained. A significant difference was found in eight procedural skills’ status between residents and faculty, though none of these were significant after accounting for multiple consecutive testing. However, the results were reviewed and a consensus for the procedures needed was developed through a focus group. A finalized procedural list was generated to determine: (a) the minimum number of times each procedure needed to be performed by the resident before deemed competent; (b) the level of competency for each procedure for respective year of residency.

**Conclusion:**

We conclude that the opinion of both the residents and the faculty as key stakeholders is vital to determine the number of procedures to be performed during an Internal Medicine Residency. Documentation of procedural competency development during the training would make the system more objective and hence reproducible. A log book was designed consisting of minimum number of procedures to be performed before attaining competency.

**Electronic supplementary material:**

The online version of this article (doi:10.1186/s12909-015-0457-4) contains supplementary material, which is available to authorized users.

## Background

Internal Medicine Residency Programs are responsible for identifying and implementing the requirements to ensure comprehensive training of the residents enrolled in the program. This includes procedural skills training, and the mandate for a competency-based postgraduate medical training requires all residency programs to teach clinical skills formally [1, 2]. Studies have shown that physicians report the procedure skills learned during the residency as the most important skills which have helped them in their career [3, 4]. Also, as depicted in one study, prospective residents prefer programs with more procedural training [5] and defining standards has been shown to be challenging yet beneficial for both patients and the physicians [6, 7]. Many physicians could depend on specialists to perform a procedure, but keeping in mind the unavailability of such expertise or inability of patients to move to such a setting frequently necessitates the Internist to perform these procedures, and even learn or master these procedures on their own due to lack of sophisticated training facilities for formal postgraduate training [8–10], hence the job of devising a comprehensive program is all the more important.

The residency program needs to identify specific procedures in which competency is to be expected of the graduating residents as a response to its specific context, and furthermore determine after performing what number of procedures under supervision are the residents deemed to be competent enough to perform them independently. However, determination of such numbers remains a challenge despite work being done in the past to unveil this dilemma [8, 11, 12]. Furthermore, experience of programs differs according to the their health care settings and requirements, and while some work from the developed world is available on the issue of imparting procedural skills competency to residents, the developing world still lags behind in its assessment of this issue.

We, at the Aga Khan University have taken the systematic instructional design process which has the core elements of analysis, design, development, implementation and evaluation (ADDIE). Instructional design is a set of procedures for developing education and training programs in a systematic, reliable and consistent manner.

The Aga Khan University Hospital (AKUH), Karachi, Pakistan is a major tertiary care hospital catering to more than 18 million people of Karachi and the surrounding region. With an operational strength of 545 beds, the facility serves over 42,000 inpatients and over 500,000 outpatients annually. Established since 1985, it is one of the few teaching hospitals in South Asia accredited by the Joint Commission for International Accreditation [13].

The Internal Medicine residency program at AKUH, comprising a total of 50 residents, is a 4 year program during which residents rotate through General Internal Medicine as well as all other medicine sub-specialties. The faculty members for the department have received training from programs in the United States of America, the United Kingdom as well as Pakistan.

### Objective

This study was conducted to identify the number of times a procedure needs to be performed by residents at different procedure status levels, during a residency program in the developing world, in order for them to achieve sufficient competency in their technical performance. We aim to formulate a set of guidelines to be implemented in our institute, as well as having applicability internationally, in our region and beyond.

## Method

This was an explanatory sequential mixed methods design [14–16], where we had collected quantitative and qualitative information sequentially. We had first collected quantitative data through cross-sectional study and then qualitative data to help refine the quantitative results, so that the study design should capture the best of both quantitative and qualitative data. We obtained quantitative data from questionnaires filled out by the faculty and the residents, and then elaborated on these findings through in-depth qualitative exploration focus group discussions (Additional file 1).

A written informed consent for participation in the study was obtained from the participants after explaining the research study and design to them.

In order to identify the optimum number of procedures required to achieve procedural skills competency a comprehensive list of procedures was identified according to the program’s contextual and certification requirements, which included peritoneal paracentesis, pleural paracentesis, urethral catheterization, lumbar puncture, CVP/JO Cath insertion into femoral vein, CVP/JO CATH insertion into Internal Jugular vein, CVP insertion into subclavian vein, Internal Jugular Vein, Temporary Pace Maker placement, Arterial Line placements, drawing of Arterial Blood gases (ABGs), Cardiopulmonary resuscitation (CPR), Bone Marrow aspiration, Joint aspiration, Chest Tube insertion, Endotracheal intubation, Swan Ganz catheterization, Pericardial paracentesis and Pleural biopsy. This list was then sent to all faculty members of the department as well as all residents enrolled in the program. The list was designed in the form of a questionnaire in order to yield two different pieces of information from faculty and residents separately: (1) what the residents and faculty felt was the adequate number of times each procedure listed is to be performed in order to achieve competency and (2) the status of the individual resident while performing the procedure in their opinion in the residency program. Four different statuses were determined as follows:

### Procedure status definitions

**Observer status:** Procedure observed without any active involvement in the intervention.

**Assistant status:** Assisted the procedure which was performed by a trained Post Graduate/Faculty

**Performed under supervision:** Performed procedure under direct supervision of a trained Post Graduate/Faculty

**Independently performed:** Perform a particular procedure independently, in consultation with the Faculty/Consultant

#### Statistical analysis

All results from the above mentioned questionnaire were compiled and analyzed using SPSS Ver. 17.0 (SPPS Inc, Chicago, IL). Basic descriptive statistics (medians and interquartile ranges) were generated. The Kolmogorov-Smirnov test identified that the data was non-parametric; hence, the Mann-Whitney test was used to compare differences in the responses of the faculty and the residents at significance level of .05. Due to multiple Mann-Whitney tests applied, a Bonferroni correction was applied to the significance level to deal with the potential problems with an inflated Type I error. Results were tabulated for presentation.

### Focus group discussions

The preliminary responses were gathered and discussed within a focus group comprising of 12 faculty members, including the program director and coordinator of Internal Medicine residency program, ex-Program Director of internal medicine, faculty representatives of all the medical sub-specialties, and two Chief Residents of Internal Medicine. The chief residents represent the opinion of residents while the sub specialty faculty provided the faculty’s perspective ensuring equal and unbiased viewpoint from all the stakeholders. Furthermore, a list of certain basic procedures (e.g. ECG recording, venupuncture, proctoscopy etc.), which were not included in the questionnaire, and certain advance procedures (e.g. Upper and Lower GI Endoscopy etc.) which were supposed to be only observed or assisted by the residents, was finalized.

This group evaluated the responses of the faculty and residents, and was given the responsibility to approve the optimal number of procedures, the year in residency when the procedure must be performed and procedure status of the residents for different procedural skills in light of the earlier conducted survey. Furthermore, wherever there was a significant difference in the opinion of the faculty and residents regarding specific procedures, the focus group gave its expert opinion regarding the final number of procedures to be recommended in the program guidelines. These consensus guidelines for the program were then approved by the Chair of the Department of Medicine and the Quality Improvement Committee of the hospital chaired by the Medical Director of the hospital. The compiled results after thorough debate and consensus were given the form of a log book to facilitate documentation and evaluation of procedures performed by a resident (Additional file 2).

### Ethical approval

Ethical approval was obtained from the Aga Khan University’s Ethics Review Committee.

## Results

A total of 64 responses were obtained from the questionnaire, in which 44 were residents while 20 were faculty members. Table 1 lists the median (and IQR) of the numbers of each procedure as suggested by both the faculty and residents. It also lists the differences between each of these observations according to p-value generated through the Mann-Whitney test, as well as the overall medians of faculty and residents for each procedure.

**Table 1:** No. of procedures required to be completed according to the responses of faculty and residents with differences according to *p*-value

	Faculty	Residents	Difference	Overall
	*n* = 20	*n* = 44	*p*-value	*n* = 64
	Median (IQR)	Median (IQR)		Median (IQR)
Mean age (SD)	43.2 (9.03)	28.5 (3.00)		33.4 (9.23)
Peritoneal paracentesis				
Observer	3 (2)	5 (2)	0.39	4 (2)
Assistant	3 (3)	4 (3)	0.86	4 (3)
Supervised	5 (5)	4 (3)	0.33	4.5 (3)
Independent	6.5 (7.5)	10 (15)	0.29	10 (12.5)
Pleural paracentesis				
Observer	4 (2)	4 (3)	0.76	4 (3)
Assistant	4 (3)	4 (4)	0.17	4 (3)
Supervised	5 (6.5)	4 (3)	0.07	5 (3)
Independent	9 (5)	6 (5)	0.51	9 (5)
Pericardial paracentesis				
Observer	4 (3)	3 (3)	0.43	4 (3)
Assistant	3 (3)	4 (3)	0.98	3 (3)
Supervised	2.5 (4)	4 (3)	0.62	4 (3)
Independent	5 (3.5)	4 (9)	0.59	5 (8)
LP				
Observer	4 (2)	5 (2)	0.53	5 (2)
Assistant	4 (3)	5 (3)	0.95	4 (3)
Supervised	4 (5)	5 (3)	0.58	5 (3)
Independent	8 (15)	5 (7.5)	0.24	7 (10)
CVP-Femoral V.				
Observer	4 (2)	4 (3)	1.00	4 (2)
Assistant	3 (2.75)	4 (2)	0.77	4 (2)
Supervised	5 (5)	4 (2.5)	0.73	5 (2)
Independent	5 (5)	5.5 (5)	0.71	5 (5)
CVP-internal jugular V				
Observer	4 (2)	4.5 (2)	0.67	4 (2)
Assistant	4 (2)	4 (2)	0.91	4 (2)
Supervised	5 (5)	4 (2)	0.13	4 (2)
Independent	5 (6.75)	5 (6)	0.75	5 (6)
CVP-Subclavian				
Observer	4 (1.75)	5 (2)	0.98	4 (2)
Assistant	4 (2)	4 (2)	0.51	4 (2)
Supervised	5 (4)	4 (2)	0.02	4 (2)
Independent	5 (7)	5 (2)	0.64	5 (3.5)
Jo Cath				
Observer	4 (2)	4 (2)	0.63	4 (2)
Assistant	4.5 (2.25)	4.5 (1.25)	0.68	5 (2)
Supervised	5 (4.25)	5 (2)	0.29	5 (1)
Independent	5 (6.5)	5 (6)	0.96	5 (6)
TPM				
Observer	4 (2)	3 (4)	0.68	3 (3.25)
Assistant	3 (4)	3 (3)	0.43	3 (3)
Supervised	5 (2)	2 (3)	0.08	3 (3)
Independent	4.5 (4.5)	3 (3)	0.72	3 (3)
Arterial line				
Observer	4 (2.75)	3 (3)	0.73	3 (3)
Assistant	3.5 (3)	3 (3)	0.47	3 (3)
Supervised	4 (3)	3.5 (3)	0.48	4 (3)
Independent	5 (2)	5 (7)	0.62	5 (7)
ABG				
Observer	4 (3)	5 (3)	0.32	5 (3)
Assistant	5 (3)	5 (2)	0.50	5 (3)
Supervised	5 (6.5)	5 (6)	0.65	5 (7)
Independent	10 (11.25)	20 (35)	0.00	20 (18.75)
CPR				
Observer	5 (2.75)	5 (5)	0.02	5 (7)
Assistant	5 (6.5)	5 (5)	0.14	5 (6.75)
Supervised	5 (5.75)	10 (5)	0.26	6 (5)
Independent	10 (15)	20 (12.5)	0.03	20 (10)
Bone marrow aspiration				
Observer	3.5 (3)	5 (2.75)	0.37	4 (3)
Assistant	4 (2.75)	4.5 (2)	0.83	4 (3)
Supervised	5 (4.5)	5 (4)	0.64	5 (3)
Independent	6 (5)	6 (10)	0.94	6 (10)
Joint aspiration				
Observer	3 (3)	2 (1)	0.06	2 (1)
Assistant	2.5 (3)	2 (1.5)	0.04	2 (1)
Supervised	4.5 (3.75)	2 (2)	0.00	3 (3)
Independent	5 (4.5)	2 (4)	0.23	5 (4)
Chest intubation				
Observer	3 (3)	3 (3)	0.96	3 (3)
Assistant	3 (3.5)	3 (3)	0.35	3 (3)
Supervised	4.5 (3.25)	4 (2)	0.34	4 (2)
Independent	5 (7)	5 (5)	0.44	5 (6)
Endotracheal intubation				
Observer	4.5 (2)	5 (2)	0.84	5 (2)
Assistant	4 (5.75)	5 (2)	0.87	5 (2)
Supervised	5 (6)	5 (4)	0.37	5 (7)
Independent	7 (7)	5 (8)	0.55	5 (7)
Swan ganz catheterization				
Observer	4 (4.25)	2 (2)	0.05	3 (2)
Assistant	2.5 (3.75)	2 (2)	0.30	2 (2)
Supervised	4.5 (3.25)	3 (3)	0.25	4 (3)
Independent	5 (5)	2 (3.25)	0.57	3 (4)
Urethral catheterization				
Observer	5 (3)	5 (2)	0.34	5 (3)
Assistant	5 (3)	5 (0)	0.06	5 (3)
Supervised	5 (6.25)	10 (6)	0.50	5 (7)
Independent	10 (34)	25 (10)	0.08	20 (21)
Pleural biopsy				
Observer	3 (3)	2 (1)	0.23	2.5 (3)
Assistant	3 (3)	2 (1)	0.03	2 (2.25)
Supervised	5 (5)	3 (3)	0.01	3 (3)
Independent	5 (6)	3 (4)	0.13	4.5 (4)

In general, similar responses were obtained from both faculty and residents for each procedure. Statistically significant differences were seen only for CVP insertion into subclavian vein (supervised), blood drawing for arterial blood gases (independent), cardiopulmonary resuscitation (observed and independent), joint aspiration (assisted and supervised), and pleural biopsy (assisted and supervised). None of these results were however significant with a Bonferroni correction applied which reduced the p-value to 0.0007. Table 2 summarizes the above mentioned procedures, depicting responses of faculty, residents, their overall median (and IQR), as well as the subsequent focus group recommendations for these procedures. Focused group was ultimately responsible for generating the final number of procedures in light of the suggestions provided by the stake holders. For example, the faculty and residents suggested that pericardial paracentesis be performed independently by residents as depicted in Table 1. However, after careful consideration by the focus group members, involved in post graduate medical education, and with the consent of chief residents it was decided that such a procedure be performed only under supervision of cardiologist and not by internal medicine residents alone irrespective of year of training.

**Table 2:** Procedures with significant differences in faculty and resident responses along with focus groups recommendations

Procedure (Status)	Faculty	Residents	Difference	Overall	Focus group recommendations
	*n* = 20	*n* = 44	*p*-value*	*n* = 64	
	Median (IQR)	Median (IQR)		Median (IQR)	
CVP-Subclavian					
Supervised	5 (4)	4 (2)	0.02	4 (2)	4
ABG					
Independent	10 (11.25)	20 (35)	0.00	20 (18.75)	20
CPR					
Observer	5 (2.75)	5 (5)	0.02	5 (7)	5
Independent	10 (15)	20 (12.5)	0.03	20 (10)	10
Joint aspiration					
Assistant	2.5 (3)	2 (1.5)	0.04	2 (1)	2
Supervised	4.5 (3.75)	2 (2)	0.00	3 (3)	3
Pleural biopsy					
Assistant	3 (3)	2 (1)	0.03	2 (2.25)	4
Supervised	5 (5)	3 (3)	0.01	3 (3)	4

All the results depicted in Table 1 were subsequently discussed under the focus group; Table 3 summarizes the final focus group recommendations about the number of times the procedures needed to be performed in order to achieve competency at different procedure status and the minimum residency level when these competencies should be obtained.

**Table 3:** Procedural skills required to be completed according to status and program level as determined by the consensus of focus group

Procedure status	Minimum residency level	Number required
1- Peritoneal paracentesis		
Observer status	PGY 1	4
Assistant status	PGY 1	4
Performed under supervision	PGY 1	4
Independently performed	PGY 2	12
2- Pleural paracentesis		
Observer status	PGY 1	4
Assistant status	PGY 1	4
Performed under supervision	PGY 1	4
Independently performed	PGY 2	12
3- Urethral catheterization		
Observer status	PGY 1	3
Assistant status	PGY 1	3
Performed under supervision	PGY 1	5
Independently performed	PGY 1	10
4- Lumbar puncture		
Observer status	PGY 1	3
Assistant status	PGY 1	6
Performed under supervision	PGY 1	4
Independently performed	PGY 2	6
5- CVP/Jo cath - femoral vein		
Observer status	PGY 1	4
Assistant status	PGY 1	4
Performed under supervision	PGY 2	4
Independently performed	PGY 2/3	5
6- CVP-internal jugular vein		
Observer status	PGY 1	4
Assistant status	PGY 1	4
Performed under supervision	PGY 2	4
Independently performed	PGY 2/3	5
7- CVP-subclavian vein		
Observer status	PGY 1	4
Assistant status	PGY 1	4
Performed under supervision	PGY 2	4
Independently performed	PGY 2/3	5
8- Jo Cath- internal jugular vein		
Observer status	PGY 1	4
Assistant status	PGY 1	4
Performed under supervision	PGY 2	5
Independently performed	PGY 2/3	5
9- Temporary pace maker placement		
Observer status	PGY 1	3
Assistant status	PGY 2	3
Performed under supervision	PGY 3	2
Independently performed	-	0
10- Arterial line placement		
Observer status	PGY 1	3
Assistant status	PGY 2	3
Performed under supervision	PGY 2	4
Independently performed	PGY 2/3	5
11- Arterial blood gases		
Observer status	PGY 1	2
Assistant status	PGY 1	4
Performed under supervision	PGY 1	5
Independently performed	PGY 1	20
12- Cardio pulmonary resuscitation		
Observer status	PGY 1	5
Assistant status	PGY 1	5
Performed under supervision	PGY 1	8
Independently performed	PGY 1	10
13- Bone marrow aspiration		
Observer status	PGY 1	4
Assistant status	PGY 1	4
Performed under supervision	PGY 11/2	5
Independently performed	PGY 12	6
14- Joint aspiration		
Observer status	PGY 1	2
Assistant status	PGY 1	2
Performed under supervision	PGY 2/3	3
Independently performed	PGY 4	2
15- Chest intubation		
Observer status	PGY 1	3
Assistant status	PGY 1	3
Performed under supervision	PGY 2	4
Independently performed	PGY 3	3
16- Endotracheal intubation		
Observer status	PGY 1	5
Assistant status	PGY 1	5
Performed under supervision	PGY 1/2	5
Independently performed	PGY 2	5
17- Swan ganz catheterization		
Observer status	PGY 1	2
Assistant status	PGY 2	2
Performed under supervision	PGY 3	2
Independently performed	-	0
18- Pericardial paracentesis		
Observer status	PGY 1	3
Assistant status	PGY 1/2	3
Performed under supervision	PGY 3	1
Independently performed	-	0
19- Pleural biopsy		
Observer status	PGY 1	4
Assistant status	PGY 2	4
Performed under supervision	PGY 2	4
Independently performed	-	1

## Discussion

While identification of specific procedures and number of times they need to be repeated to achieve a level of competency is a matter of debate [12, 17], methods to determine this optimal set of numbers are also contentious. Expert consensus guidelines, although widely reported in literature [6, 11, 12, 18–22], have been questioned due to their inherent subjectivity [18, 23, 24], leading to a need for more standardized and vigorous system [23, 25]. Setting these criteria and standards has been shown to positively impact training of post graduate trainees [6, 26, 27]. Furthermore, when these recommendations are exposed to formal testing in terms of the skills imparted, these numbers may not seem sufficient to impart the competence in procedures deemed generally advanced and specialized [17, 24]. This, however may not always be the case, especially in the more routine procedures of the internal medicine residency training [12].

Therefore, while developing these guidelines, equal weightage was given to the residents’ and the faculty’s opinion to arrive at the optimal number, while the focus group served to streamline their opinions in cases where the opinions diverged significantly. Taking residents’ opinion to form these tools has been suggested by earlier literature [28–30]. Lack of funding and resources even at places with specialized training programs have been identified as possible causes for inadequate procedural training [31]. We must come up with ways to overcome these hurdles and by making this tool we have tried, at least in part, to increase the competence of our trainees, while using the limited recourses available to us.

Residents in our country have to undergo a mandatory internship year before joining a residency program and have already given the first part of their accreditation exam (Fellowship of College of Physicians and Surgeons—FCPS); they are adequately exposed to the ground realities of training in order to give them an informed opinion. Taking responses from faculty is more intuitive as these are the ones who perform these procedures themselves or supervise and train others.

The focus group was necessary to factor in the expectations from individuals who have actually designed or are responsible for academic and administrative affairs of the program. The individuals comprised, thus remained aware of the realities of the society in which graduates of the program are expected to serve. Hence, their expert consensus was important in bringing the expectations of the patients and society into account while designing these guidelines. This system evokes experience published for other systems and programs such as that of the Accreditation Council for Graduate Medical Education (ACGME) of the United States [32] translated in our particular context.

We also believe that supervision and assessment at different competence levels or procedure status levels for necessary skill acquirement could have better objectivity, than only direct observation to acquire competency [12, 33]. Furthermore, this may serve to limit the traditional “see one, do one, teach one” model which has been called into question due to the inherent risks of complication and incompetence associated with it [18, 21], while not resorting to the over use of simulations and models in resource poor settings.

We, at the Aga Khan University have a trainee centered program and strongly believe in the transition of trainer based to trainee based curriculum (Table 4). We train our residents in areas they feel they need most assistance and where they have lagged in their previous years during undergraduate or postgraduate training. It must also be kept in mind that none of the curriculum development is done without a rigorous evaluation of suggestions that are put forward by the residents, as done in this exercise of log book development. To put it aptly, we direct and not dictate the training of our residents, keeping in mind the changing trends and upcoming need of skills in a physician.

**Table 4:** Procedures in which performance competency is required by the Aga Khan University, Karachi, Pakistan

Procedures	AKU guidelines
Peritoneal paracentesis	X
Pleural paracentesis	X
Urethral catheterization	X
Lumbar puncture	X
CVP/Jo Cath femoral vein	X
CVP/Internal jugular vein	X
CVP/Subclavian vein	X
Jo Cath internal jugular vein	X
Temporary pacemaker	X^a^
Arterial line and blood drawing	X
Arterial blood gases	X
Cardiopulmonary resuscitation	X
Bone marrow aspiration	X
Joint aspiration	X
Chest intubation	X
Endotracheal intubation	X
Swan-ganz catheterization	X
Pericardial paracentesis	X^b^
Pleural biopsy	X
Recording and reporting ECGs	X^c^
Venupuncture	X^c^
Nasogastric tube placement	X^c^
Proctoscopy	X^d^
Renal biopsy	X^d^
Lower GI endoscopy/sigmoidoscopy	X^d^
Upper GI endoscopy/sigmoidoscopy	X^d^
Peritoneal dialysis	X^d^
Hemodialysis	X^d^
Bronchoscopy	X^d^
ETT	X^d^
Abdominal ultrasound	X^d^

^a^Specific method not specified

^b^Not to be performed independently

^c^Basic procedure for which no. not determined

^d^Only observed and assisted status

**Fig. 1:**
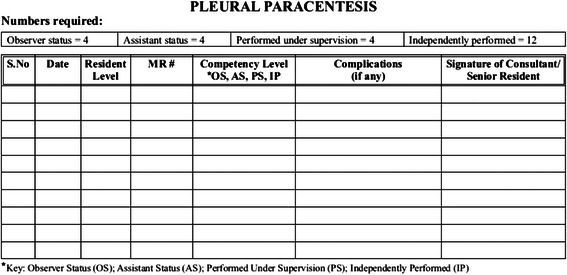
Sample logbook page

As a result of this exercise, a log book has been designed, wherein the residents are required to log all procedures performed during each residency year. Figure 1 shows a sample page of the log book. Table 4 lists the names of the procedures performed by residents at the Aga Khan University. This will facilitate in reporting complications, if any, encountered during or after the performance of the procedure. This is to ensure that optimal recommendations could be arrived at after field testing the new recommendations, while also serving the long term aims of improving the practices of post graduate medical education for our institutional program.

The development of this log book has been placed at the “implementation” element of the ADDIE model as explained earlier. We have analyzed the need and importance of the issue, designed and developed a log book which is currently being filled by the residents at their respective levels of training. We believe that this tool will help bring a uniform consistency and competency to the training of the resident, which unfortunately has not been achieved earlier [24]. The following part of the study will be the evaluation part where we would be able to determine the number of procedures performed and competency achievement.

### Study limitation

Firstly, the initial survey was performed on the faculty and residents of only one institution, further multi-centered studies must be performed in order to generalize its applicability. Secondly, time for competency level accomplishment may vary according to residents’ rotation, personal motivation, availability of cases and their learning abilities.

We feel that one potential bias could be information bias as data obtained was subjective and dependent upon the individual faculty/residents’ understanding of obtaining expertise in a certain procedure.

Future studies done on the same topic may rectify the number of procedures to be performed by residents to become competent in a particular procedure. Log book is designed in such a way that a resident is being observed through various stages and finally performs supervision.

It will be valuable to also see which procedures are performed and also the attainment of competence in performing these procedures in programs globally and compare the training methodologies between the programs in this part of the world to those in the West.

The ultimate value of these set of numbers compiled in a log book lies in the cumulative effort and input of the residents and faculty of a teaching hospital to define competence of a trainee in procedural skills in the Internal Medicine Residency Program. These numbers can never be of any value unless practically implemented, monitored and regularly updated.

## Conclusion

It has remained a challenge to identify the precise number and procedures to achieve procedural skill competence in an Internal Medicine Residency Program. It is vital to consider the opinions of all stakeholders including both the post graduate trainees and faculty before any guidelines are formulated. Documentation of each skill developed and accountability for each mistake made during the training would make the system more objective and hence reproducible globally. A general consensus should be sought to eliminate the difference of region or country where the training is provided regarding Procedural Skills Competency.

This study suggests that residency programs in different parts of the world have different requirements regarding procedural skills. It also adds to the literature in terms of an illustrative exercise for development of guidelines in a developing world setting, which is responsive to its national healthcare context. Further assessment of the logbook developed will benefit in streamlining these proposed guidelines and may serve as a model for other programs in similar settings. These can thus form the basis and provide the tools for conducting, potentially large scale, multicenter studies to promulgate a set of such precise guidelines, having a much wider applicability.

Through this study, we have identified different ‘numbers’ of procedures and their respective status quantitatively, and compiled them in a log book form. Further work is required to fine tune this effort and to add a qualitative aspect to determine the efficiency and effectiveness of a resident while performing, assisting or even observing these procedures. Ultimately, the next step for the University is to evaluate how this documentation of acquisition of procedural skills can change the quality of care in terms of fewer complications due to appropriate supervision and better skills thus enriching the quality of residents we produce and eventually impact patient care.

## Additional files

Additional file 1: **Questionnaire.** (DOCX 78 kb) Additional file 2: **Log book page. Figure S1.** (DOC 130 kb)
