# Supplementary material for: Optimum number of procedures required to achieve procedural skills competency in internal medicine residents
Source: BMC Med Educ. 2015 Oct 23;15:179. doi: 10.1186/s12909-015-0457-4 (PMC4619250; doi:10.1186/s12909-015-0457-4)
Supplement: Additional file 1: — Questionnaire. (DOCX 78 kb) [file 12909_2015_457_MOESM1_ESM.docx]

**SAMPLE LOGBOOK PAGE**

**Description:**

**This is a sample page of the logbook designed to document procedural competency in Internal Medicine Residents.**
