# Supplementary material for: Optimum number of procedures required to achieve procedural skills competency in internal medicine residents
Source: BMC Med Educ. 2015 Oct 23;15:179. doi: 10.1186/s12909-015-0457-4 (PMC4619250; doi:10.1186/s12909-015-0457-4)
Supplement: Additional file 2: — Log book page. Figure S1. (DOC 130 kb) [file 12909_2015_457_MOESM2_ESM.doc]

Questionnaire

Name (Optional) _____________ Gender: Male / Female Age: _______

Title: Faculty/Resident

Position (Faculty): Assistant Professor/Associate Professor/Professor

Position (Resident): R1/R2/R3/R4

Please specify how many times the following clinical procedures, should be done during

Post-graduate training by the residents for each level of Residency: Please find below the definitions of each level of competency for your reference.

Observer status: Procedure observed without any active involvement in the intervention.

Assistant status: Assisted the procedure which was performed by a trained Post Graduate/ Faculty

Performed under supervision: Performed procedure under direct supervision of a trained Post Graduate/Faculty

Independently performed: Perform a particular procedure independently, in consultation with the Faculty/Consultant

Please write the number against each year of residency and level of competency.

| PROCEDURE | COMPETENCY LEVEL | | | | |
| --- | --- | --- | --- | --- | --- |
|  | Observer  Status | Assistant  Status | Performed Under Supervision | Performed Independently | |
| Peritoneal paracentesis |  |  |  |  | |
|  |  |  |  |  |  |
|  |  |  |  |  |  |
|  |  |  |  |  |  |
| Pleural paracentesis |  |  |  |  | |
|  |  |  |  |  |  |
|  |  |  |  |  |  |
|  |  |  |  |  |  |
| Pericardial paracentesis |  |  |  |  | |
|  |  |  |  |  |  |
|  |  |  |  |  |  |
|  |  |  |  |  |  |
| Lumber puncture |  |  |  |  | |
|  |  |  |  |  |  |
|  |  |  |  |  |  |
|  |  |  |  |  |  |
| Insertion of CVP line from femoral vein |  |  |  |  | |
|  |  |  |  |  |  |
|  |  |  |  |  |  |
|  |  |  |  |  |  |
| Insertion of CVP line from internal jugular vein |  |  |  |  | |
|  |  |  |  |  |  |
|  |  |  |  |  |  |
|  |  |  |  |  |  |
| Insertion of CVP line from subclavian vein |  |  |  |  | |
|  |  |  |  |  |  |
|  |  |  |  |  |  |
|  |  |  |  |  |  |
| Insertion of Jo Cath |  |  |  |  | |
|  |  |  |  |  |  |
|  |  |  |  |  |  |
|  |  |  |  |  |  |
| Temporary Pacemaker insertion |  |  |  |  | |
|  |  |  |  |  |  |
|  |  |  |  |  |  |
|  |  |  |  |  |  |
| Arterial Line Insertion |  |  |  |  | |
|  |  |  |  |  |  |
|  |  |  |  |  |  |
|  |  |  |  |  |  |
| Drawing Arterial Blood Gases |  |  |  |  | |
|  |  |  |  |  |  |
|  |  |  |  |  |  |
|  |  |  |  |  |  |
| Cardio pulmonary Resuscitation |  |  |  |  | |
|  |  |  |  |  |  |
|  |  |  |  |  |  |
|  |  |  |  |  |  |
| Bone marrow aspiration & trephine |  |  |  |  | |
|  |  |  |  |  |  |
|  |  |  |  |  |  |
|  |  |  |  |  |  |
| Joint aspiration |  |  |  | |  |
|  |  |  |  |  |  |
|  |  |  |  |  |  |
|  |  |  |  |  |  |
| Chest intubation |  |  |  | |  |
|  |  |  |  |  |  |
|  |  |  |  |  |  |
|  |  |  |  |  |  |
| Endotracheal Intubation |  |  |  | |  |
|  |  |  |  |  |  |
|  |  |  |  |  |  |
|  |  |  |  |  |  |
| Swan Ganz Catheter Insertion |  |  |  | |  |
|  |  |  |  |  |  |
|  |  |  |  |  |  |
|  |  |  |  |  |  |
| Urethral Catheterization |  |  |  | |  |
|  |  |  |  |  |  |
|  |  |  |  |  |  |
|  |  |  |  |  |  |
| Pleural Biopsy |  |  |  | |  |
|  |  |  |  |  |  |
|  |  |  |  |  |  |
|  |  |  |  |  |  |
